# Memory-enhancing treatments reverse the impairment of inhibitory avoidance retention in sepsis-surviving rats

**DOI:** 10.1186/cc7103

**Published:** 2008-10-28

**Authors:** Lisiane Tuon, Clarissa M Comim, Fabrícia Petronilho, Tatiana Barichello, Ivan Izquierdo, João Quevedo, Felipe Dal-Pizzol

**Affiliations:** 1 Laboratório de Neurociências, Programa de Pós-Graduação Ciências da Saúde, Unidade Acadêmica de Ciências da Saúde, Universidade do Extremo Sul Catarinense, Av. Universitária, 1105, 88806-000 Criciúma, SC, Brasil; 2 Laboratório de Fisiopatologia Experimental, Programa de Pós-Graduação em Ciências da Saúde, Unidade Acadêmica de Ciências da Saúde, Universidade do Extremo Sul Catarinense, Av. Universitária, 1105, 88806-000 Criciúma, SC, Brasil; 3 Centro de Memória, Instituto de Pesquisas Biomédicas, Pontifícia Universidade Católica do Rio Grande do Sul, Av Ipiranga, 6690, 90610-000 Porto Alegre, RS, Brasil

## Abstract

**Introduction:**

Survivors from sepsis have presented with long-term cognitive impairment, including alterations in memory, attention, concentration, and global loss of cognitive function. Thus, we evaluated the effects of memory enhancers in sepsis-surviving rats.

**Methods:**

The rats underwent cecal ligation and perforation (CLP) (sepsis group) with 'basic support' (saline at 50 mL/kg immediately and 12 hours after CLP plus ceftriaxone at 30 mg/kg and clindamycin at 25 mg/kg 6, 12, and 18 hours after CLP) or sham-operated (control group). After 10 or 30 days, rats were submitted to an inhibitory avoidance task. After task training, animals received injections of saline, epinephrine, naloxone, dexamethasone, or glucose. Twenty-four hours afterwards, animals were submitted to the inhibitory avoidance test.

**Results:**

We demonstrated that memory enhancers reversed impairment in the sepsis group 10 and 30 days after sepsis induction. This effect was of lower magnitude when compared with sham animals 10 days, but not 30 days, after sepsis.

**Conclusions:**

Using different pharmacologic approaches, we conclude that the adrenergic memory formation pathways are responsive in sepsis-surviving animals.

## Introduction

Central nervous system dysfunction secondary to sepsis can occur in 8% to 70% of septic patients [1]. In addition, it has been demonstrated that survivors from sepsis presented long-term cognitive impairment, including alterations in memory, attention, concentration, and global loss of cognitive function [2]. However, the mechanisms associated with these alterations are still unclear. We had previously demonstrated that sepsis survivors after 10 and 30 days of cecal ligation and perforation (CLP) presented memory impairment and behavior alterations, and we proposed this model as a useful tool to determine the mechanisms associated with long-term cognitive impairment in sepsis survivors [3-7].

Aversively motivated learning is influenced by neuromodulators and hormones related to emotional aspects of the training experience. Emotionally arousing events cause a release of epinephrine (EPI) and an increase in corticosterone, and both EPI and corticosteroids are known to modulate memory [8]. Other systems could modulate the formation of emotionally motivated memory. For example, opioid receptors are involved in memory modulation, and post-training injections of the opioid antagonist naloxone (NAL) enhance retention of inhibitory avoidance in rats [9]. In this context, we investigated whether some of the molecular mechanisms associated with memory formation are preserved in sepsis survivors using the post-training administration of EPI, NAL, dexamethasone (DEX), and glucose (GLU) in a step-down inhibitory avoidance task in rats.

## Materials and methods

### Animals

Two hundred forty adult male Wistar rats (220 to 300 g) were obtained from our breeding colony. They were housed five to a cage with food and water available *ad libitum *and were maintained on a 12-hour light/dark cycle (lights on at 7 a.m.). Behavioral procedures were conducted between 8 a.m. and noon. All experimental procedures involving animals were performed in accordance with the National Institutes of Health *Guide for the Care and Use of Laboratory Animals *[10] and the Brazilian Society for Neuroscience and Behavior (SBNeC) recommendations for animal care, and approval for the study was given by the ethics committee from our university.

### Cecal ligation and perforation surgery

Animals were subjected to CLP as described [11] with adaptations [12-14]. Briefly, rats were anesthetized with a mixture of ketamine (80 mg/kg) and xylazine (10 mg/kg) given intraperitoneally. Under aseptic conditions, a 3-cm midline laparotomy was performed to allow exposure of the cecum with the adjoining intestine. The cecum was tightly ligated with a 3.0 silk suture at its base, below the ileocecal valve, and was perforated once with a 14-gauge needle. The cecum was then gently squeezed to extrude a small amount of feces from the perforation site returned to the peritoneal cavity, and the laparotomy was closed with 4.0 silk sutures. Animals were resuscitated with normal saline (50 mL/kg subcutaneously) immediately and 12 hours after CLP. All animals were returned to their cages with free access to food and water. In the sham-operated group, the rats were submitted to all surgical procedures but the cecum was neither ligated nor perforated. After surgery, the sepsis group received 'basic support' (30 mg/kg ceftriaxone and 25 mg/kg clindamycin subcutaneously every 6 hours for a total of 3 days). The sham-operated group received the volume of saline corresponding to antibiotic administration. Survival rates were 100% in the sham group and 47% in the sepsis group, which were in accordance with our previous reports [12-14]. Animals were randomly distributed to sham and CLP groups and to memory enhancers or saline, and 10 or 30 days after surgery the animals underwent an inhibitory avoidance test.

### Inhibitory avoidance

The inhibitory avoidance procedure was described in a previous report [15]. The apparatus was an acrylic box (50 × 25 × 25 cm) whose floor consisted of parallel-caliber stainless-steel bars (1 mm diameter) spaced 1 cm apart, and a platform that was 7 cm wide and 2.5 cm high. Animals were placed on the platform and their latency to step down on the grid with all four paws was measured with an automatic device. Training sessions were performed 10 or 30 days after surgery. Immediately after stepping down on the grid, animals received a foot shock of 0.3 mA and 2 seconds. In test sessions carried out 24 hours after training, no foot shock was given and the step-down latency (maximum of 180 seconds) was used as a measure of retention. The behavioral tests were performed by the same person that was blind to the experimental group.

### Intervention

The animals were divided in groups of 15 and received an intraperitoneal injection of saline (control), EPI (25 μg/kg), NAL (0.4 mg/kg), DEX (0.3 mg/kg), or GLU (320 mg/kg) immediately after training. The doses were selected based on a previous report [15].

### Statistical analyses

Data for inhibitory avoidance are presented as the median (interquartile range) of retention test latencies. Differences between training and test session latencies within each group were determined using the Wilcoxon test. The Kruskal-Wallis test was performed in comparisons between groups. For comparisons between various treatments, the Mann-Whitney test with the Bonferroni adjustment modified by Finner was used.

## Results

**Figure 1:**
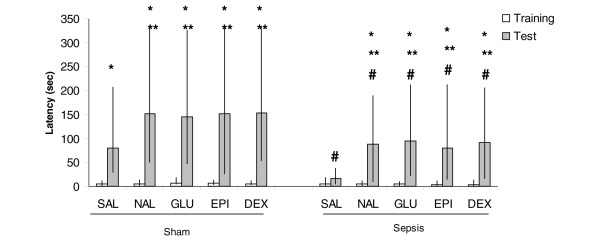
Inhibitory avoidance task 10 days after cecal ligation and perforation (CLP). Animals were submitted to CLP or were placed in a sham-operated group. Ten days after surgery, animals underwent the training test for an inhibitory avoidance task. Immediately after training, animals received a single injection of saline (SAL), epineohrine (EPI), naloxone (NAL), dexamethasone (DEX), or glucose (GLU), and animals were tested 24 hours later. Data are presented as median (interquartile range) of retention test latencies. *Significantly different between training and test, *P *< 0.05, Wilcoxon test. **Significantly different between NAL, GLU, EPI, or DEX and SAL in the test section, *P *< 0.05, Mann-Whitney test (Kruskal-Wallis chi-square 13.4, *P *= 0.009). ^#^Significantly different between sham and CLP in the test section, *P *< 0.05, Mann-Whitney test (Kruskal-Wallis chi-square 27.48, *P *< 0.001).

**Figure 2:**
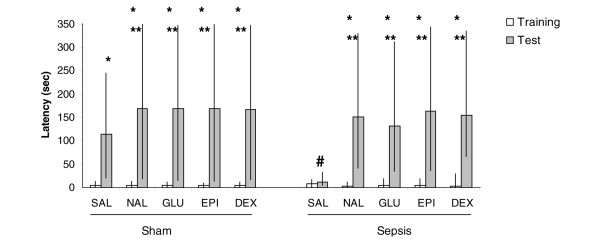
Inhibitory avoidance task 30 days after cecal ligation and perforation (CLP). Animals were submitted to CLP or were placed in a sham-operated group. Thirty days after surgery, animals underwent the training test for an inhibitory avoidance task. Immediately after training, animals received a single injection of saline (SAL), epineohrine (EPI), naloxone (NAL), dexamethasone (DEX), or glucose (GLU), and animals were tested 24 hours after. Data are presented as median (interquartile range) of retention test latencies. *Significantly different between training and test, *P *< 0.05, Wilcoxon test. **Significantly different between NAL, GLU, EPI, or DEX and SAL in the test section, *P *< 0.05, Mann-Whitney test (Kruskal-Wallis chi-square 27.7, *P *< 0.001). ^#^Significantly different between sham and CLP in the test section, *P *< 0.05, Mann-Whitney test (Kruskal-Wallis chi-square 30.8, *P *= 0.001).

As expected, classical memory enhancers EPI (Z = -3.05, *P *= 0.002 for both 10 and 30 days after surgery, comparing training and test), NAL (Z = -3.06, *P *= 0.002 for both 10 and 30 days after surgery, comparing training and test), DEX (Z = -3.05, *P *= 0.002 for both 10 and 30 days after surgery, comparing training and test), or GLU (Z = -3.06, *P *= 0.002 for both 10 and 30 days after surgery, comparing training and test) improved memory in the inhibitory avoidance 10 and 30 days after sham surgery (Figures 1 and 2). Ten days after CLP, EPI (Z = -3.18, *P *= 0.001, comparing training and test), NAL (Z = -3.06, *P *= 0.002, comparing training and test), DEX (Z = -3.06, *P *= 0.002, comparing training and test), or GLU (Z = -3.06, *P *= 0.002, comparing training and test) reversed memory impairment but this effect was of lower magnitude when compared with sham animals (Figure 1) (Additional data file 1). In addition, memory enhancers reversed memory impairment 30 days after sepsis induction in the same magnitude when compared with sham animals (Z = -3.18, *P *= 0.001 to EPI, comparing training and test; Z = -3.06, *P *= 0.002 to NAL, comparing training and test; Z = -2.93, *P *= 0.003 to DEX, comparing training and test; Z = -3.06, *P *= 0.002 to GLU, comparing training and test) (Figure 2) (Additional data file 1).

## Discussion

The present study demonstrated that the administration of memory enhancers (EPI, NAL, DEX, or GLU) in sepsis survivors reverses long-term cognitive impairment. These results suggest that, instead of the demonstrated neuronal loss after sepsis [16], the molecular mechanisms associated with affective memory formation are preserved in sepsis survivors. The effect of cognitive enhancers seemed to be of a different magnitude 10 or 30 days after sepsis, suggesting that the mechanisms responsible for affective memory formation were more compromised early after sepsis recovery. This observation is consistent with our previous results that demonstrated a time-dependent recuperation of memory deficits in sepsis-surviving rats [3-7]. We had previously demonstrated that survivors from sepsis presented habituation and non-aversive and aversive memory deficits [3-7], but the results presented here are limited to aversive (affective) memory, which has several characteristics that are very different than declarative, procedural, or instrumental memory [17].

Several studies have found alterations in neurocognitive function following critical illness [18-23], and recognition of these long-term sequelae in survivors from critical illnesses has shifted outcome values from reduction in hospital mortality to patient-centered outcomes [24]. However, to date, the mechanisms associated with these alterations are still unclear; thus, animal models can be used to address these limitations. Explicitly or implicitly, learning tasks in animals involve the performance or the inhibition of some form of movement in response to sensory or other cues. Of the various training procedures used, perhaps the most popular in the past few years have been the Morris water maze, one-trial inhibitory avoidance, and various forms of fear conditioning, all of which closely mimic human situations of daily life. The inhibitory avoidance task relies heavily on the dorsal hippocampus but also depends on the entorhinal and parietal cortex and is modulated by the amygdale [25,26]. In this way, we believe that our results, using the CLP model, provide relevant insights into the mechanisms involved in the cognitive deficits associated with sepsis and into therapeutic approaches to this problem.

None of the neuropsychological tests that are used in humans, however, assessed memory of this sort. Rather, cognitive assessments evaluated patients on measures of, for example, declarative memory and working memory. Recognition of objects is thought to be a critical component of human declarative memory that is mainly dependent on the hippocampus. Object recognition is commonly impaired in human patients affected by neurodegenerative diseases or who have suffered brain injury [27,28]. In addition, executive functions are mediated by independent and interacting neural systems that may be compromised by different forms of pathology, leading to a range of cognitive profiles. The frontostriatal network mediates those cognitive functions that are needed to optimize performance in complex tasks and that include a number of psychological processes. Recognition memory was previously demonstrated to be altered in animal models of sepsis [5], but there were no published data that assessed executive memory. Therefore, future animal studies that test the effect of critical illness on cognitive functions should employ outcomes that assess functioning in homologous systems in animals involving the frontal lobe and/or hippocampus as is observed in humans.

All the used memory enhancers seemed to exert their effect by modulating the adrenergic system, and there is evidence that catecholamine has profound effects on cognitive function [29]. Immediate post-training systemic injections of EPI or norepinephrine enhance the consolidation and/or storage of novel information in rats [29]. The enhancing effects of glucocorticoids on memory consolidation depend on the integrity of the amygdala noradrenergic system [30] as do the enhancing effects of NAL [31]. The effects of the noradrenergic system on memory formation seemed to be dependent on GLU since a noradrenergic agonist enhances memory formation by facilitation of GLU uptake at the time of memory consolidation [32]. These effects are not restricted to animal models. Recent evidence indicates that EPI enhances memory consolidation in humans [33]. In addition, it is now well established that glucocorticoid hormones enhance memory consolidation [34] and that GLU modulates memory formation in humans [35]. Opioid peptides mediate alterations in human memory during emotional states and help to explain why memories may be selectively deficient under conditions of stress [36]. Thus, since survivors from the intensive care unit presented long-term cognitive impairment, including alterations in memory, and this was associated with a decrease in quality of life [23], our results brings the perspective to improve long-term outcome in sepsis survivors.

Some limitations of our study must be pointed out. First, septic animals in comparison with sham controls received antibiotics, which could have neuroprotective properties [37]. We had demonstrated previously that the antibiotics used in our model did not modify memory performance in our model [4]; thus, we believe that this limitation is of minor importance. Second, it would be interesting to examine the effects of other drugs that are more promising as clinically useful cognitive enhancers (that is, rolipram) [38], but since this is the first demonstration of enhancing memory after CLP, we decided to use more 'classical' memory enhancers. Third, only single doses of the memory enhancers were evaluated; thus, instead of a normal response observed using these doses, we could not rule out the possibility that in sepsis survivors the dose response curve to these enhancers may be altered. Fourth, we demonstrated that sepsis altered memory of an emotional event (that is, foot shock). One may suggest that the response to a new stimulus depends on the intensity of a previous emotional challenge and that we are not observing a true sepsis effect, but a procedure-related effect. We tried to avoid this limitation by randomly dividing animals between groups, and animals were subjected to the same surgical procedure, with sepsis being the sole difference between groups. There are also some clues that suggest that animals are similar in regard to stress response. First, in the open-field task, there were no differences in the number of crossings and rearings between groups in the training session, demonstrating no difference in motor and exploratory activities between groups [3,4], and stressed animals presented alterations in the exploratory activity [39]. Second, in analyses at 10 and 30 days after CLP, sham and septic animals presented no differences in foot shock sensitivity as assessed by the 'flinch and jump' response test [40].

## Conclusion

We demonstrated, for the first time, using different pharmacologic approaches, that the adrenergic system is responsive in sepsis-surviving animals in different intensities 10 and 30 days after sepsis. Since this system is relevant to memory formation in humans and animals, our results brings the perspective that the modulation of the adrenergic system could be a suitable tool in the treatment of memory deficits observed in sepsis survivors.

## Key messages

• The administration of memory enhancers in sepsis survivors reverses long-term cognitive impairment.

• The adrenergic system could be pharmacologically modulated in sepsis-surviving animals being a target in the future treatment of cognitive impairment in sepsis survivors.

## Abbreviations

CLP: cecal ligation and perforation; DEX: dexamethasone; EPI: epinephrine; GLU: glucose; NAL: naloxone.

## Competing interests

The authors declare that they have no competing interests.

## Authors' contributions

LT conceived of this study, participated in the design of the study, and drafted the manuscript. CMC, FP, and TB participated in the design of the study and performed experimental analyses. II, JQ, and FD-P participated in the design of the study and drafted the manuscript.

## Supplementary Material

Additional file 1 The following additional data are available with the online version of this paper. Additional data file 1 is a table showing the absolute retention values for each group. Click here for file
