# Supplementary material for: Memory-enhancing treatments reverse the impairment of inhibitory avoidance retention in sepsis-surviving rats
Source: Crit Care. 2008 Oct 28;12(5):R133. doi: 10.1186/cc7103 (PMC2592772; doi:10.1186/cc7103)
Supplement: Additional file 1 — The following additional data are available with the online version of this paper. Additional data file 1 is a table showing the absolute retention values for each group. [file cc7103-S1.doc]

10 days

| Groups and Treatments | Absolute values Training  Median (25th-75th) | Absolute values Test  Test  Median (25th-75th) | Z-values between training/test |
| --- | --- | --- | --- |
| Sham + Saline | 4,5 (3-6,75) | 79,5 (50,75-126,50) | 0,002 |
| Sham + EPI | 6,0 (2,7-7) | 151,5 (125,25-179,75) | 0,002 |
| Sham + NAL | 4,5 (3-9) | 152,0 (102,25-180) | 0,002 |
| Sham + DEX | 5,5 (2,25-6) | 153,5 (100,25-178,25) | 0,002 |
| Sham + GLU | 6,5 (2-10,75) | 145,0 (98-180) | 0,002 |
| Sepsis + Saline | 5,5 (3,25-12,25) | 15,5 (10,25-22) | 0,055 |
| Sepsis + EPI | 4,0 (2-7) | 80,0 (65,50-131,50) | 0,001 |
| Sepsis + NAL | 5,0 (3,25-6,75) | 88,5 (79,50-100,25) | 0,002 |
| Sepsis + DEX | 4,0 (2,25-8,25) | 90,5 (73,50-114,75) | 0,002 |
| Sepsis + GLU | 4,5 (3-5,75) | 94,0 (71,25-117,50) | 0,002 |

30 days

| Groups and Treatments | Absolute values Training  Median (25th-75th) | Absolute values Test  Test  Median (25th-75th) | Z-values between training/test |
| --- | --- | --- | --- |
| Sham + Saline | 6,0 (4-8) | 115,0 (93-130) | 0,002 |
| Sham + EPI | 4,5 (4-6) | 170,0 (156-180) | 0,002 |
| Sham + NAL | 5,5 (5-8,25) | 169,5 (150,50-180) | 0,002 |
| Sham + DEX | 6,0 (5-7) | 168,0 (150,75-180) | 0,002 |
| Sham + GLU | 4,5 (4-7,5) | 170,0 (154,75-178,25) | 0,002 |
| Sepsis + Saline | 8,0 (6-9,5) | 11,50 (5,75-22,25) | 0,071 |
| Sepsis + EPI | 5,0 (4-15) | 180,0 (129-180) | 0,001 |
| Sepsis + NAL | 4,5 (4-15) | 131,50 (96,25-180) | 0,002 |
| Sepsis + DEX | 3,0 (2-13) | 150,0 (87-180) | 0,003 |
| Sepsis + GLU | 4,0 (2-25,75) | 155,0 (88,25-180) | 0,002 |
